# Lipid Vesicle Shape Analysis from Populations Using Light Video Microscopy and Computer Vision

**DOI:** 10.1371/journal.pone.0113405

**Published:** 2014-11-26

**Authors:** Jernej Zupanc, Barbara Drašler, Sabina Boljte, Veronika Kralj-Iglič, Aleš Iglič, Deniz Erdogmus, Damjana Drobne

**Affiliations:** 1 Seyens Ltd., Ljubljana, Slovenia; 2 University of Ljubljana, Biotechnical Faculty, Ljubljana, Slovenia; 3 University of Ljubljana, Faculty of Health Sciences, Ljubljana, Slovenia; 4 University of Ljubljana, Faculty of Electrical Engineering, Ljubljana, Slovenia; 5 Northeastern University, Boston, Massachusetts, United States of America; Temple University School of Medicine, United States of America

## Abstract

We present a method for giant lipid vesicle shape analysis that combines manually guided large-scale video microscopy and computer vision algorithms to enable analyzing vesicle populations. The method retains the benefits of light microscopy and enables non-destructive analysis of vesicles from suspensions containing up to several thousands of lipid vesicles (1–50 µm in diameter). For each sample, image analysis was employed to extract data on vesicle quantity and size distributions of their projected diameters and isoperimetric quotients (measure of contour roundness). This process enables a comparison of samples from the same population over time, or the comparison of a treated population to a control. Although vesicles in suspensions are heterogeneous in sizes and shapes and have distinctively non-homogeneous distribution throughout the suspension, this method allows for the capture and analysis of repeatable vesicle samples that are representative of the population inspected.

## Introduction

Lipid vesicle studying techniques range from visual investigation of individual vesicles to population studies of vesicles in suspension. The most common visual investigation techniques are light, fluorescence, confocal, atomic force, and electron microscopy [1]–[7]. For population studies in which average data on the entire vesicle populations are generated, the most common techniques are flow cytometry, dynamic light scattering (DLS), X-ray diffraction techniques, and others [8]–[11]. Most frequently, both approaches are required to thoroughly explore the advantages of using artificial vesicles in lipid membrane studies.

Vesicle populations are dynamic in shape, their volume changes by their nature, and they contain vesicles of different sizes and heterogeneous shapes. Vesicle shape is determined by the amphiphilic characteristics of its membrane and is subject to curvature fluctuations and spontaneous shape transformations [12]–[14]. Visual techniques reveal minute membrane curvature fluctuations of a single vesicle [1] but lack the capacity to provide information on their shapes and sizes, which are representative of the entire vesicle population. When treated, some vesicles show considerable changes in their shape, whereas others remain less affected [6]. Such dynamics of shape and size changes are difficult to follow using existing visualization approaches. Morita et al. [6] succeeded in capturing vesicle shape dynamics by observing their transformations in real time. Haluska et al. [2] also studied time scales of membrane transformations and used a fast digital camera to directly record the membrane process within the time resolution of 50 µs. Diguet et al. [7] also made time lapse observations to study the morphological dynamics of lipid vesicles. High temporal resolution allows for the depiction of minute morphological changes in individual vesicles but cannot capture the population. To study the entire population, flow cytometry is perhaps the most explored tool. Using this tool, high-dimensional quantitative measurements of light scatter and the fluorescence emission properties of hundreds of thousands of individual objects are considered in each analyzed sample [15]. Identification of population characteristics is used routinely in both research labs to study normal and abnormal structure of objects and clinical labs. However, because this approach cannot provide morphological information, visual techniques are employed to identify population characteristics.

Recently, in some studies, researchers attempted to collect the population characteristics of lipid vesicles, such as size distribution, from micrographs. Sato et al. [8] used Adobe Photoshop to manually segment vesicles from light microscopy images, and Fa et al. [16] used the Axiovision program from Zeiss. In both cases, vesicle quantities were below one hundred. Characterizing such small groups of vesicles from micrographs is possible using manual approaches [1], [17]; however, image quantification becomes increasingly difficult and tedious for larger numbers of vesicles [8]. Recently, Hermann et al. [18] used circular Hough Transform image segmentation to analyze vesicle populations; however, such segmentation assigns only circles to vesicles and is unsuitable for analyzing vesicle shapes.

An entire new perspective must be considered to enable bulk analysis of thousands of vesicles based on microscopy methods, the result of which would be comparable to the results of flow cytometry. One must employ a large-scale microscopy approach in which multiple micrographs are stitched together to allow the capture of large samples [19], [20]. Typically, this capture is achieved using an automated microscope that programmatically guides the slide to record multiple micrographs that, combined, cover a larger area of the specimen. However, when recording vesicles, the slide is most commonly guided by a technician who also manually adjusts the focal plane to the depth at which the majority of vesicles is present [21]. This procedure renders the generic large-scale microscopy approaches [22]–[25] less useful and requires customized solutions to allow a combination of large-scale microscopy with manual slide operation. Zupanc et al. [26] introduced an algorithm that enables stitching mosaics from video micrographs containing lipid vesicles, and is used for the purpose of the experiments presented in this study. Moreover, because manually segmenting thousands of vesicles is too cumbersome, an automated or semi-automated image analysis method is essential to decrease the time required for this task. Zupanc et al. [27] proposed one such algorithm, which is used to segment vesicles in this study.

The aim of this paper is to describe in detail the method that combines both the visual and the population analysis of lipid vesicles, allowing the presentation of quantified population parameters along with their visual properties. To achieve this aim, video microscopy is coupled with computer vision algorithms. This approach enables recording and analysis of samples containing up to several thousand vesicles. For each individual vesicle, its cross-section diameter and isoperimetric quotient (for a nearly spherical vesicle, this parameter is also a measure of roundness, here abbreviated as IPQ) are calculated. Size distributions of these parameters are then compared among different samples.

This study discusses the repeatability of vesicle quantities and their shapes in initial populations created through electroformation, and the repeatability of the sampling within a single population that is crucial to this method’s usability. Moreover, also of interest is how sedimentation of vesicles over time influences the sampling and after what incubation durations a representative vesicle sample may be gathered.

## Experimental

### Chemicals and Apparatus

Synthetic 1-palmitoyl-2-oleoyl-*sn*-glycero-3-phosphocholine (POPC) and cholesterol were obtained from Avanti Polar Lipids, Inc. (Alabaster, AL, USA). Stock solutions of both POPC and cholesterol (at 1 mg ml^–1^ concentration) were prepared by dissolving powder lipids in a mixture of CHCl_3_ (66%, v/v) and MeOH (33%, v/v). Sucrose and glucose were purchased from Sigma-Aldrich (Steinheim, Germany). Solutions of 0.3 M aqueous sucrose and glucose were prepared using distilled water fresh before each experiment.

All processing was performed on a PC with an 8-core i7-2600K CPU @ 3.40 GHz, 16 GB RAM running Windows 7 Professional 64-bit edition, 2009. The image processing algorithms were developed in Matlab R2011a (MathWorks, MA, USA). Microsoft Excel 2007 (Microsoft Corporation, WA, USA) and Matlab were used for statistical analysis. The invert microscope used was a Nikon Eclipse TExp.2000-S with a Sony CCD video camera module attached, model XC–77 CE, and 400× magnification was used. CoverWellTM Perfusion chambers PC4L-0.5 were from Grace Bio-Labs Sigma-Aldrich (Steinheim, Germany).

### Preparation of GUVs by Electroformation

The GUVs were prepared using a modified electroformation method from saturated and unsaturated lipids [28] from POPC and cholesterol, combined in a ratio of 4∶1 (v/v). The lipid mixture solution (40 µL) was spread over two platinum electrodes and the solvent was allowed to evaporate for 2 h *in vacuo.* The coated electrodes were then placed 4 mm apart in an electroformation chamber containing 2 mL of 0.3 M sucrose solution. An alternating electric field of magnitude 5 V/mm and frequency of 10 Hz was applied to the electrodes for 2 h. Then, the magnitude and frequency of the alternating electric field were gradually reduced at intervals of 15 min, first to 2.5 V/mm and 5 Hz, then to 2.5 V/mm and 2.5 Hz, and finally to 1 V/mm and 1 Hz. In a single experiment (three were conducted and are presented in this paper), all vesicles were taken from the same electroformation chamber. After the electroformation, 0.3 M sucrose solution containing electroformed GUVs was added to 0.3 M glucose solution in a ratio 3∶5 (v/v). The vial was inverted five times to obtain equal density of the vesicles throughout the volume. The resulting vesicles consisted of a 4- to 5-nm thick lipid bilayer and had an average diameter of 6–8 µm.

**Figure 1:**
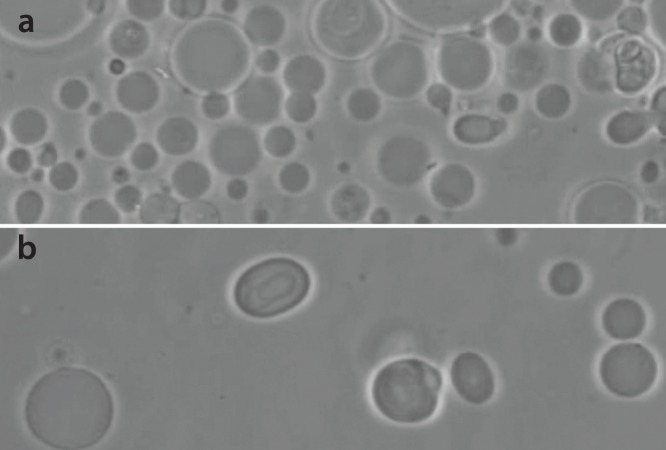
Micrographs of two vesicle populations with different vesicle densities in solution. Vesicle abundance is higher in (a) than in (b). The lower abundance of vesicles (b) is preferred for better performance of the computer vision algorithms.

To enable computerized vesicle segmentation from micrographs, the vesicles in the solution should not be too densely grouped together (Figure 1a). The vesicle solution was diluted to achieve lower abundance per volume (Figure 1b). In our case, the solution was diluted 1∶6; however, because the abundance of vesicles prepared by the aforementioned preparation method varies, this dilution ratio might not be correct for every case. To achieve the appropriate abundance (Figure 1b is a visual reference), the dilution ratio must be determined during each individual experiment.

### Exposure Experiments and Imaging

**Figure 2:**
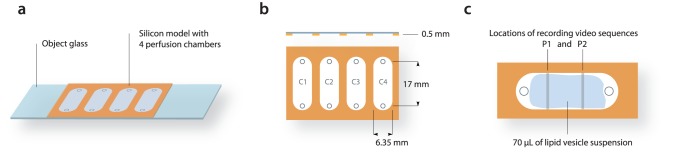
Scheme of the perfusion chambers (a). Silicone model with four perfusion chambers named C1–C4 (b). The side view is given in the figure and the depth of the chambers is 0.5 mm. A single chamber with two tracks (P1 and P2) locations of two tracks recorded in each chamber (c). The two tracks together cover approximately 3% of the chamber area.

The vesicle suspension, which is prepared using the same electroformation process, is referred to as a single population for the purpose of the statistical analysis. We transferred 70 µL droplets of vesicle suspension to each of the four perfusion chambers (Figure 2a Figure 2b) using 20–200 µL Pippete (Eppendorf, Hamburg, Germany). In some experiments, droplets of the same vesicle suspension were transferred to up to eight chambers. This step marks the beginning of the exposure experiment. After predefined exposure durations (in our experiments these durations were all within the first hour of incubation), samples of every chamber were recorded.

Two video tracks were recorded in each chamber, and these two together are referred to as one sample. In Figure 2c, the locations at which the videos were recorded are marked as P1 and P2, and they were recorded in a one-dimensional movement. Each video track was approximately 2 minutes long (∼3,000 frames), with a per pixel spatial resolution of 0.25 µm (video image resolution of 570×762 pixels). A single video covers a two-dimensional area of 1.15±0.05 mm^2^. With two such tracks, approximately 3% of the chamber area is recorded. Recording a single chamber sample (two video tracks) takes up to 4 minutes.

### Data Processing and Analysis

**Figure 3:**
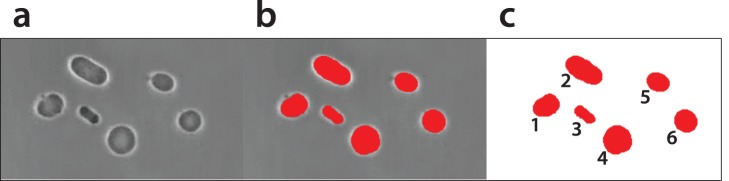
Light micrograph of vesicles in a micrograph (a), vesicles with overlaid segmentation (b), mask with segmented vesicles separated from the background in which each vesicle is marked with a number (c).

This step consists of video processing, image processing, and statistical data analysis. Each video of a single track was stitched into a single large image – a mosaic [26] the resulting mosaic was approximately 20.000×762 pixels, where one pixel relates to 0.25 µm of the specimen. The vesicles in these mosaics were segmented with a custom developed algorithm [27] based on a Markov random field image segmentation model. The result of this segmentation is a binary mask (Figure 3), where all vesicles are marked with a single color and the background is removed.

To assure accuracy of the results, an expert subsequently examined and corrected all automatically segmented vesicles. In addition to the vesicles, the medium sometimes also contains crystallized lipids that, in some cases, resemble vesicles, making them difficult to distinguish using automated segmentation alone. At the current quality of automatic segmentation, this step consumed approximately 20 minutes of the expert’s time per sample.

### Vesicle Shapes and Statistical Analysis

Different vesicle shape parameters may be extracted from images containing vesicle masks (Figure 3c). Two most interesting vesicle population dynamics for analysis are vesicle size and a measure of a vesicle’s roundness. Both are computed from the mask created using image segmentation of vesicles from micrographs. The effective diameter of a vesicle cross-section from the vesicle area in pixels is estimated and presented in µm. As a measure of roundness, we use an estimate of the isoperimetric quotient (4 * π * Area/Perimeter∧2), which is the area divided by the perimeter squared and normalized by 4π to ensure that a circular vesicle has an isoperimetric quotient of 1 [29]. The value of the quotient decreases toward zero as the shape diverges from a perfect circle.

## Results

We conducted three experiments (Exp. 1 to Exp. 3) in which vesicles in suspension were incubated for up to 1 hour. At multiple times (exact numbers in Table 1), the vesicles were sampled by recording videomicroscopy sequences that were later analyzed using computer vision algorithms to count the vesicles and calculate their sizes and isoperimetric quotients.

**Table 1:** Vesicle measurements from three experiments; the experimental parameters for each experiment (time is the duration of incubation at recording; the number of chambers is the same as the number of samples).

Experiment	Time [min]	Number ofChambers	Quantity:Mean ± Std	QuantityRSD	Diameter [µm]Mean ± Std	DiameterRSD	IPQMean ± Std	IPQ RSD
Exp.1	3	8	775±45	6%	11.4±0.4	3%	.92±.00	0%
	30	8	2221±157	7%	8.5±0.4	4%	.93±.00	0%
	3	4	590±47	8%	7.7±0.3	4%	.93±.02	2%
Exp.2	30	4	1837±78	4%	6.6±0.2	3%	.92±.02	2%
	60	4	2446±96	4%	6.3±0.1	2%	.93±.01	1%
Exp.3	20	4	497±34	6%	6.5±0.3	4%	.98±.01	1%
	50	4	844±35	4%	5.7±0.2	3%	.99±.01	1%

Relative standard deviation (RSD) measures the variation between chambers of the same populations. Each population had multiple chambers; Table 1 contains the mean vesicle quantity per chamber mean vesicle diameter and mean vesicle isoperimetric quotient (IPQ).

### Vesicle Population Repeatability

#### a) Vesicle Quantities

**Figure 4:**
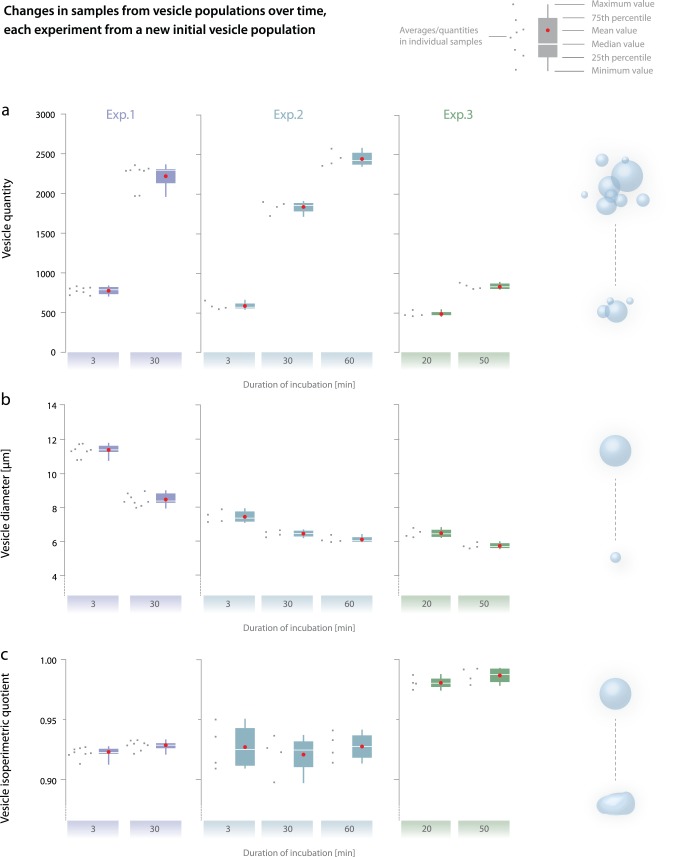
Changes in samples from different vesicle populations over time (from 3 to 60 minutes). Each experiment was conducted using a new initial vesicle population and each sample point represents a single chamber. Vesicle quantities (a), mean projected diameter sizes (b), and mean isoperimetric quotients (c). The box plot consists of mean minimal and maximal values 25th 50th and 75th percentile.

The three experiments were conducted to test the variability among the chambers distributed from the same vesicle population (Figure 4a). The more comparable the samples are from these chambers, the more reproducible and reliable are the results. To begin with, we took the experimental setup from our preliminary experiments [30]. In Exp. 1, eight chambers of control vesicles were first recorded after 3 minutes, and then after 30 minutes of incubation. During the experiment, vesicles slowly collected in the focal plane near the bottom of the chamber attributable to gravity. We observed the increase in vesicle quantities between minutes 3 and 30 (Table 1).

To assess the effect of gravity after more than 30 minutes, in Exp. 2, the samples were recorded also after 60 minutes of incubation. In this experiment, vesicle quantity increased on average by a factor of three between minutes 3 and 30 and then increased by only a factor of 1.3 from 30 to 60 minutes. The sedimentation was not as pronounced between 30 and 60 minutes as in the first 30 minutes, suggesting that the majority of the vesicles had already settled in the first 30 minutes. Therefore, in Exp. 3, sampling after 3 minutes of incubation was abandoned and conducted after 20 and 50 minutes of incubation.

In Exp. 1 and Exp. 2, the abundance of vesicles in the chambers (up to 2,400 in an acquired sample) encumbered automatic segmentation of vesicles from micrographs. Therefore, to improve automatic segmentation from micrographs, in Exp. 3, vesicle abundance was reduced by diluting the vesicle suspension with 0.3 M glucose solution at a ratio 1∶4 (v/v), which also resulted in a decrease in the vesicle quantity by approximately four times. The lower abundance of vesicles in Exp. 3 still resulted in approximately the same relative standard deviation (RSD) between samples as in the previous two experiments. In all three experiments and for all recording times, the RSD among the samples was below 8%.

#### b) Vesicle diameter

Figure 4b provides a comparison of the vesicle diameter sample means, in each of the three experiments showed the similar trend of the vesicle diameter sample mean decreasing over time. Gravity and buoyancy explain this trend: the larger (heavier) vesicles collect at the bottom faster than the smaller ones. For the early recording times, particularly after 3 minutes, the majority of the small vesicles did not yet collect at the bottom of the chamber and were not present in the recorded sample. The differences between 20 and 50 minutes are less pronounced, indicating that the majority of the vesicles were already collected in the focal plane within the first 20 minutes.

**Figure 5:**
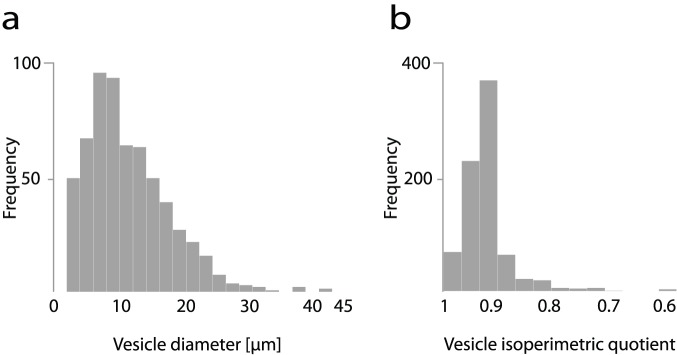
Histograms of vesicle diameter size distribution (a) and vesicle isoperimetric quotient (b). Data samples for both histograms were taken from one randomly selected sample and their shapes are representative of all samples.

The distribution of the vesicle diameter size is described as broad, mono-modal, and lognormal (Figure 5a), and all visible vesicles in the recorded samples are larger than 1 µm and up to 50 µm in diameter. Among samples from the same population, the relative standard deviation was less than 4% for all recording times.

#### c) Vesicle morphology

Figure 4c compares isoperimetric quotients of the individual samples for all three experiments. This measure of vesicle roundness distinguishes among more circular vesicles (IPQ diverges to 1) and those with more complex perimeters for which IPQ decreases. Within a single population, the relative standard deviation between samples was always 2% or less (Table 1). Figure 5b presents the IPQ distribution of a single sample. While mean vesicle IPQs in samples from Exp.1 and Exp.2 are in similar ranges, the mean IPQs in Exp.3 are significantly higher. The vesicle shapes and sizes between different initial vesicle populations have been shown to vary [34], [35] and the difference in IPQs between the three experiments can be attributed to the variability of initial populations prepared at different times following the same electroformation procedure.

## Discussion

We present a method for analyzing lipid vesicles that allows us to non-destructively conduct shape and size analysis of thousands of vesicles and simultaneously allows for visual inspection of individual ones. This method is achieved by combining operator guided large-scale light microscopy with custom computer vision algorithms. We automatically count the vesicles and compute two parameters for each vesicle: diameter of its contour (computed from cross-section area) and 2-D isoperimetric quotient (a measure of roundness of contour). The size distribution of these parameters enables a comparison of samples from the same population through time or, for example, samples of a treated population with an untreated control.

The problem with existing lipid vesicle population analysis techniques is that they either mask the heterogeneity of a population or require the vesicles to be pretreated in a specific manner to be investigated, such as adding probes to the membrane system for fluorescent imaging or using lipid-specific staining, contrast agents, or cryo-fixation to enable samples to be visualized using electron microscopy, and others. Moreover, both sample preparation and the presence of a vacuum in the EM chamber notably affect vesicle morphology [4]. The two frequently used population analysis approaches are flow cytometry and DLS. The use of DLS is more reasonable for investigation of small or large unilamellar vesicles (SUVs and LUVs) because particle/liposome size is determined in a fixed range. For example, Chen et al. [11] used DLS to study lipid vesicles with diameters in the 30–120 nm range. In the range of cell sized-liposomes, DLS is reliable only for homogenous samples, which is “*usually not very achievable*” [8].

Flow cytometry enables the inspection of cell-sized liposomes or giant unilamellar vesicles (GUVs); however, fluorescent labeling is a prerequisite for flow cytometry-based analysis to distinguish the signal from the background noise [8], [11]. Sato et al. [8] improved the performance of flow cytometry to simultaneously measure the internal aqueous volume and lipid membrane volume of heterogeneous individual cell-sized lipid vesicles with a small number of lamellae and a diameter of up to approximately 10 µm. This study improved the performance of microscopy investigation by obtaining population parameters on vesicle sizes and shapes using computer vision algorithms and, therefore, enabling visual characterization of a large number of vesicles [26], [27].

Several reasons exist for selecting 20 and 50 minutes after the beginning of vesicle incubation as the optimal times during which to record the samples. In the first minutes, the effect of gravity and buoyancy causes larger vesicles to collect at the bottom of the chamber faster than smaller ones. The reason for this sedimentation is the weight density difference between the sucrose inside the vesicles and the iso-osmolar glucose solution in the suspension [7]. Figure 4a displays this phenomenon, in which the vesicle quantity in the samples in Exp. 1 and Exp. 2 increased fourfold between 3 and 30 minutes, and less than twofold between 30 and 60 minutes. Although the exposure duration could be prolonged, in the experimental setup, the vesicle quantity starts to decrease after 60 minutes from desiccation because the chambers are not completely sealed.

When observing membrane stability in terms of fluctuations and morphological changes, other authors [6] proved that the time span of 30 minutes is adequate. This time span is also adequate for our experimental setup; a single operator can record multiple samples from multiple (for example, treated versus untreated) populations when vesicles are incubated for 20 and 50 minutes, as in Exp. 3. Recording at these two times generates information about the time-dependent shape changes in the vesicles.

In other similar studies [7], the exposure duration varied significantly. Similarly, Tomita et al. [31] performed observations after different durations that ranged from 20 minutes to 160 minutes. Diguet et al. [7] observed vesicles for 2 hours. Spontaneous giant phospholipid vesicle shape transformations in the time interval of hours were observed, revealing the existence of phospholipid membrane nanotubes [32], [33]. In other cases, some observed membrane processes required a time resolution of milliseconds [2].

The applicability of this method depends strongly on the sampling’s repeatability and representativeness. When initial vesicle populations are prepared multiple times following the same electroformation procedure, the size and shape distributions in these populations are shown to vary [34], [35]. This result suggests that each experiment must be evaluated separately. However, when a single vesicle population is sampled multiple times after the same incubation duration, the vesicle size and shape distributions are repeatable, which is most crucial for this method’s usability. Four to eight samples from the same initial population were recorded after a specific duration of incubation, and the relative standard deviations among the samples were only up to 8%. High repeatability of vesicle quantities in individual samples shows that the recorded samples are representative for the population even though the vesicles are distributed heterogeneously throughout the suspension.

Given the microscopy-based approach, the vesicles are visible and various morphological changes are observed directly [36]. The method described provides probability distributions of vesicle diameters and isoperimetric quotients (Figure 5). The data on the initial polydispersity of the vesicles in the population and their initial shape status are important before any further treatment is conducted [12]. In addition, the images of all recorded vesicles remain in an image database, allowing for the visual verification of all of the results. At a later point, if an algorithm is developed that automatically calculates other vesicle properties or, for example, classification of different vesicle morphology types, the revised computer vision algorithms may be applied to all previous experiments without needing to repeat them.

The presented method offers new opportunities in the basic research on interactions of different substances with lipid vesicles and a fast, reliable, and reproducible screening test for characterizing the size and shape diversity in lipid vesicle populations. Such tests could be significantly beneficial in research on the interactions among membranes and different agents such as toxins [37], [38], peptides [39], nanoparticles [30], and drugs [40], in which a complex and diverse mode of interaction is expected.
